# Encephalopsin (OPN3) protein abundance in the adult mouse brain

**DOI:** 10.1007/s00359-012-0754-x

**Published:** 2012-09-19

**Authors:** Juuso Nissilä, Satu Mänttäri, Terttu Särkioja, Hannu Tuominen, Timo Takala, Markku Timonen, Seppo Saarela

**Affiliations:** 1 Department of Biology, University of Oulu, P.O. Box 3000, 90014 Oulu, Finland; 2 Institute of Health Sciences, University of Oulu, Oulu, Finland; 3 Institute of Diagnostics, Forensic Medicine, University of Oulu, Oulu, Finland; 4 The National Institute for Health and Welfare, Oulu, Finland; 5 Institute of Diagnostics, Pathology, University of Oulu, Oulu, Finland; 6 Department of Pathology, Oulu University Hospital, Oulu, Finland; 7 Oulu Health Centre, Oulu, Finland

**Keywords:** Cerebellum, Cerebral cortex, Circadian entrainment, Extraretinal phototransduction, Hypothalamus

## Abstract

Encephalopsin belongs to the family of extraretinal opsins having a putative role in CNS tissue photosensitivity. Encephalopsin mRNA has earlier been localized in rodent brains, but expression and localization of the protein has not yet been reported. In this study, we aimed to define encephalopsin protein abundance and localization in the rodent brain. The distribution and localization of encephalopsin protein in a mouse brain and selected peripheral tissues were analysed in ten mice, using Western blotting and immunohistochemistry. The specificity of immunoreaction was validated by primary antibody omitting and immunizing peptide blocking experiment. We found encephalopsin protein abundant in the mouse brain, but not in the periphery. Encephalopsin protein was present in neurons of the mouse cerebral cortex, paraventricular area, and cerebellar cells. Our results show that encephalopsin is expressed at the protein level in different brain areas of the mouse. Therefore, the suggested idea that encephalopsin plays a role in non-visual photic processes seems to be applicable. Evidently, further investigations are needed to find out the signalling mechanisms, and the potential physiological role of encephalopsin in phototransduction due to the changes in ambient light.

## Introduction

Encephalopsin, also called OPN3 or panopsin (Halford et al. 2001), belongs to one encephalopsin/tmt-opsin subfamily of the seven subfamilies of the opsins. Opsins are known to mediate phototransduction in both visual and non-visual systems by being transmembrane proteins acting as G-protein-coupled receptors (GPCRs) (Terakita 2005). Even though the exact function of encephalopsin has remained largely unknown, it has been suggested to play a role in non-visual photic processes, such as the entrainment of circadian rhythm or the regulation of pineal melatonin production (Kasper et al. 2002; White et al. 2008).

Visible ambient light is capable of penetrating to the mammalian brain (Ganong et al. 1963), and several studies show extravisual opsin genes expressed in mammalian brain at mRNA-level (Halford et al. 2001; Kasper et al. 2002; Tarttelin et al. 2003; White et al. 2008; Allen Institute for Brain Science 2011). However, the actual distribution and functional role of extravisual opsins in participating neural actions and CNS tissue regulation calls for investigation.

Wide distribution of encephalopsin mRNA was first shown by Blackshaw and Snyder (1999) in the mouse brain. Recently, encephalopsin/OPN3 gene expression in cerebellar compartments was observed in distinct lobules and Purkinje cells as revealed by three-dimensional reconstruction of in situ hybridization data (Lein et al. 2007). It was also shown that the expression of the OPN3 gene in the cerebellum is quite coherent, involving sagittal bands of expression but also sharply delineated diagonal bands lacking OPN3 mRNA (Lein et al. 2007).

In addition to measurements of mRNA expression, quantifications of protein expression are also needed in order to increase our understanding of the role of OPN3 at tissue and cellular level. The aim of the present study was to analyse the expression, relative amount, and distribution of the OPN3 protein in selected brain areas and peripheral tissues of the adult mouse. To the best of our knowledge, no previous studies exist on the distribution of OPN3 protein in the mouse brain.

## Methods

### Sampling and analyses

**Fig. 1:**
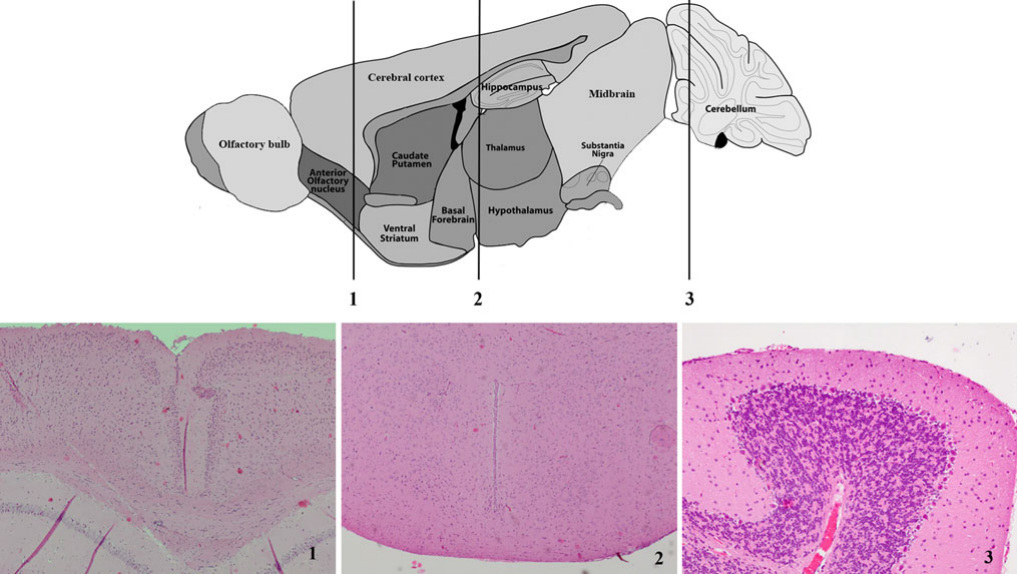
Representative paraffin **(**Mayer’s hematoxylin and eosin stain) sections through cerebral cortex (**1**), paraventricular area (PVA) (**2**), and cerebellum (**3**) along with corresponding anatomical brain atlas. The orientation of the sections is transversal

Ten male Balb/c mice, 8 weeks of age, *m* = 23.7 ± 1.1 g, were euthanized by carbon dioxide and sacrificed by cervical dislocation. The central nervous system tissue samples collected were cerebral cortex (grey matter), hypothalamus as localized by recognizing paraventricular area (PVA), and cerebellum (Fig. 1). Additionally, peripheral tissues investigated were liver, kidney, skeletal muscle (*m. rectus femoris*), cardiac muscle, adrenal gland, testis, ovary from three female mice, and plasma.

### SDS-PAGE and Western blotting

The tissue samples were homogenized (Tissue Lyser Qiagen, Retsch) in 6 vol of homogenization buffer (62.5 mM Tris–HCl, pH 6.8) including protease inhibitors (leupeptin 1 μg mL^−1^; pepstatin 1 μg mL^−1^; PMSF 1 mM) and heated in a sample buffer as previously described in Laemmli (1970). SDS-PAGE was performed using a 12.5 % separating gel (EZ-Run Protein Gel Solution, Fisher Bioreagents, UK). Each sample contained 2.11 μg of protein determined by the Bradford (1976) method. The proteins were electrophoretically separated at 150 V for 80 min. The separated proteins were electroblotted to a nitrocellulose membrane according to the method of Towbin et al. (1979). Membranes were incubated for 2 h in primary antibody (rabbit polyclonal to encephalopsin, cat # ab75285, Abcam, UK) and with secondary antibody (blotting grade affinity purified, AP-conjugated goat anti-rabbit, Bio-Rad, USA) for 2 h. Antibody detection was performed with bromo-4-chloro-3-indolyl phosphate mono-(–toluidinium) salt/nitro blue tetrazolium (BCIP/NBT) substrate. The dilution for the primary antibody was 1:500. The optical densities of the detected bands were analysed with FluorS MultiImagerTM program (Bio-Rad, USA). In order to determine the specificity of the band observed, control experiments were included where (1) the primary antibody was omitted (no primary control), or (2) an immunizing peptide blocking experiment (negative control) was performed with a fivefold excess of immunizing peptide (sequence LDVHGLGCTVDWKSKDANDSSFVLFLFLGC, United Peptide Co, USA) relative to primary antibody, i.e., 10 μg ml^−1^.

### Immunohistochemical (IHC) staining

Immunohistochemistry was performed on blocks of frozen tissues cut into 8 μm sections with a cryostat microtome at −25 °C. The sections were mounted on APES coated slides, fixed with pre-cooled ethanol and blocked with 10 % goat normal serum with 1 % BSA in TBS. Encephalopsin antibody (rabbit polyclonal to encephalopsin, Abcam, UK) was used at a final concentration of 2 μg mL^−1^. Sections were incubated overnight at 4 °C. Fluorescent dye labelled secondary antibody (concentration 10 μg mL^−1^, Alexa Fluor goat anti-rabbit IgG, Invitrogen, USA) was used with 1 h incubation in RT, and the samples were cover-slipped with mounting medium (ProLong Antifade, Molecular Probes, USA). The images of the sections were obtained with a confocal laser scanning microscope (LSM-5 Pascal, Zeiss, Germany) by using excitation at 488 nm. Control sections were incubated in TBS with 1 % BSA devoid of primary antibody. An immunizing peptide blocking principal with neutralized primary antibody was also used for IHC with a fivefold excess of immunizing peptide.

## Results

**Fig. 2:**
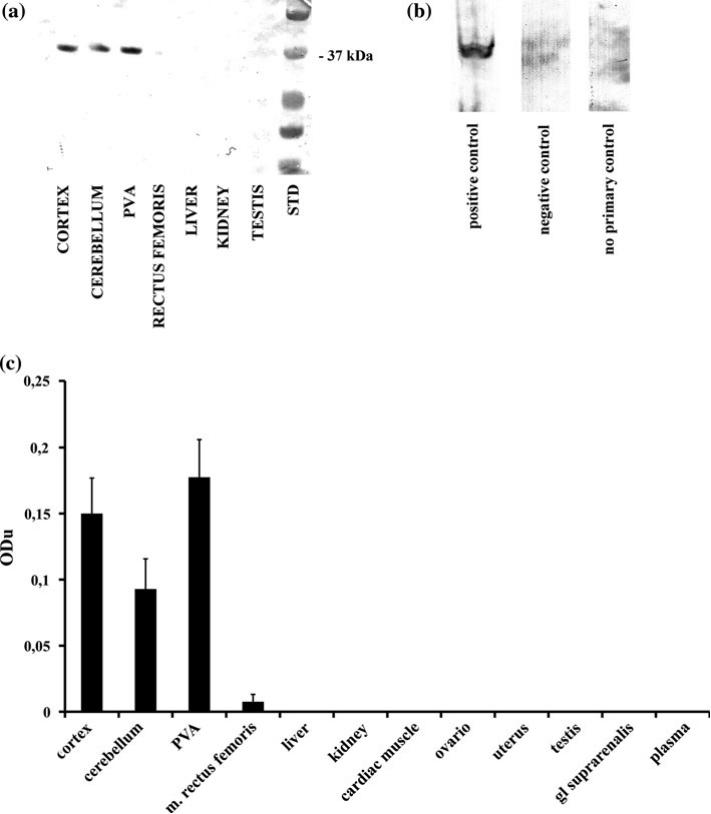
Expression of OPN3 in different tissues of mouse. **a** A representative from Western blot membrane showing expression of OPN3. std: precision plus protein standard mass ladder; **b** method controls: retina as positive control, PVA as negative control with antibody preincubated with the immunizing peptide, PVA as no primary control; **c** A bar graph summarizing results from Western blot analysis showing mean optical density (ODu) ± SE of OPN3 in tissue homogenates processed for immunoblotting (*N* = 10)

Western blot was used in order to analyze the abundance and relative amount of OPN3 in the cerebral cortex, PVA, and cerebellum (Fig. 2 a). Comparison of the relative densities of the encephalopsin bands indicated no significant differences between the specific brain areas. However, the amount of OPN3 in PVA was 1.2-fold compared with the cerebral cortex, and 1.9-fold compared with the cerebellum (Fig. 2 c). Western blots detected a single band of approximately 45 kDa, consistent with predicted protein size for encephalopsin. No protein bands were detected in the control samples devoid of primary antibody. Furthermore, the staining was completely prevented by co-incubation with the immunizing peptide. Mouse retina was used as a positive control showing a single band of 45 kDa (Fig. 2 b, see Halford et al. 2001).

**Fig. 3:**
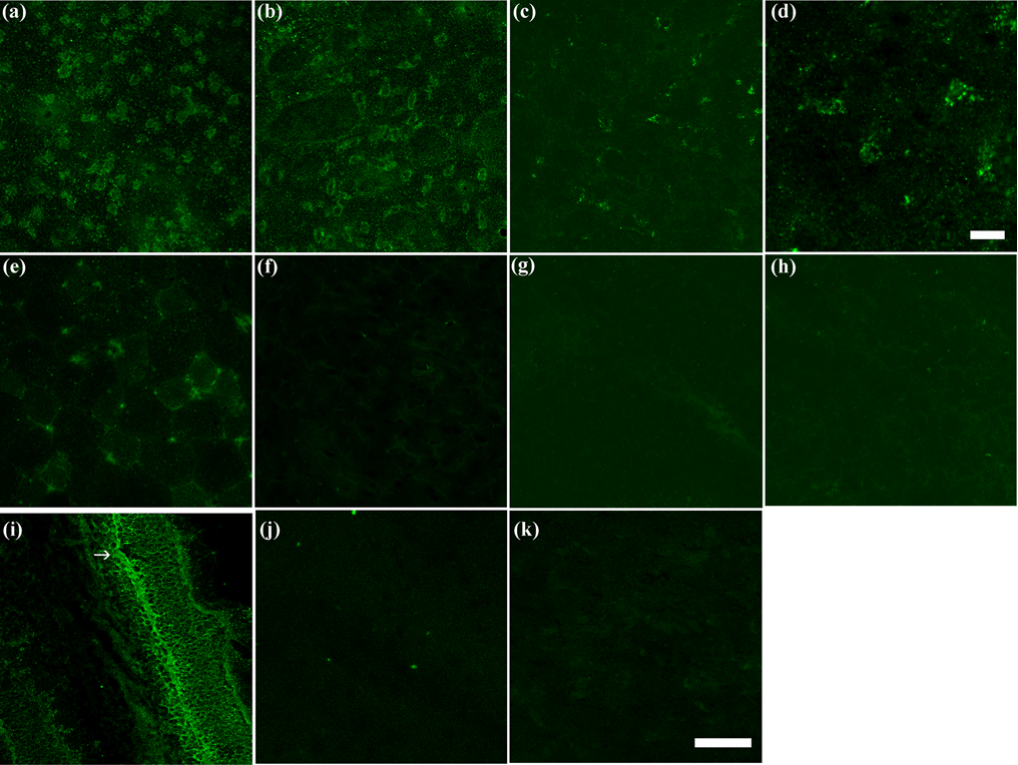
OPN3 expression in different brain areas and peripheral tissues of mouse. Representative results of immunofluorescence staining. **a** cerebral cortex, **b** paraventricular area (PVA), **c** cerebellum, **d** neuron in cerebellar tissue, **e** rectus femoris muscle, **f** liver, **g** testis, **h** kidney. Controls: **i** retina (positive control) where ganglion cell layer (indicated with *arrow*) is seen immuno-positive, **j** negative control of retina with antibody preincubated with the immunizing peptide **k** no primary control of retina. *Bar* 50 μm (**a–c**; **e–k**), 10 μm (**d**)

**Fig. 4:**
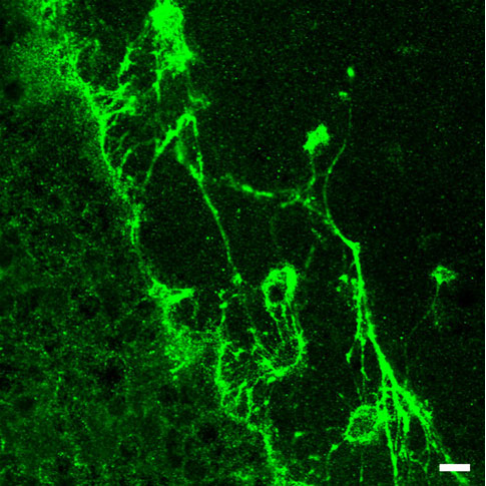
OPN3 expression in large single neurons with complex dendritic branching between the outer and inner layer of cerebellar cortex. *Bar* 10 μm

In order to indicate the localization of OPN3, immunofluorescence staining was used, revealing punctate or granular immunoreaction of variable intensity for OPN3. According to the results, OPN3 was present in most neurons of the cerebral cortex (Fig. 3 a), paraventricular area (PVA, Fig. 3 b), and in the cerebellum (Figs. 3 c, d), especially in cerebellar cells (Fig. 4). No expression of OPN3 was seen in neuronal nuclei, glial cells, or neuropil. *Musculus rectus femoris* of only two mice out of ten showed OPN3 at the periphery of muscle fibres (Fig. 3 e). Other peripheral tissues studied, such as cardiac muscle, liver (Fig. 3 f), testis (Fig. 3 g), kidney (Fig. 3 h), and adrenal gland, were immunonegative. Ovary and uterus were also tested as negative in three females (data not shown). Mouse retina was used as a positive control (Fig 3 i). Negative control sections of retina with antibody preincubated with the immunizing peptide or devoid of primary antibody (no primary control) showed no staining (Fig. 3j, k).

## Discussion

To the best of our knowledge, this was the first study to report the expression and localization of OPN3 protein in the adult mouse brain. Earlier, OPN3 has been observed only at an mRNA detection level in the mouse brain (Blackshaw and Snyder 1999). However, mRNA-level expression of genes does not inherently mean there is existing protein (Greenbaum et al. 2003). Our results are in agreement with the findings of Blackshaw and Snyder (1999) showing a strong specific binding of the OPN3 antibody on wide areas of the mouse cerebral cortex, PVA, and cerebellum.

The role of OPN3 in mammalian brain functions has until now remained elusive. The role of OPN3 is most likely related into its phylogenetic background as an extraretinal ciliary phototransductive membrane protein (Shichida and Matsuyama 2009). Indeed, the capability of opsin proteins to adopt their functional phototransductive role when expressed on extra-visual neurons is shown in studies, where foreign species’ opsin genes are added to neurons via viral vectors (Boyden et al. 2005; Warren et al. 2006). In these studies, opsin-mediated phototransduction has been confirmed by electrical intracellular recordings during illumination. From that point of view, it is reasonable to hypothesize that endogenous opsins will also carry their functional role as a part of extravisual phototransduction. In general, all known vertebrate photoreceptors use an opsin protein bound to a vitamin A-chromophore as photopigment. Over species and opsins, the form of used vitamin-A (retinal) varies, but the principle is always the same: when the photon is absorbed by the retinal chromophore, this molecule isomerizes from 11-*cis*-retinal form to the all-*trans*-retinal form. This conformational change allows the intracellular terminus of opsin to trigger a G-protein cascade leading into a change in receptor membrane potential. The cascade converting photic energy into neural responses is called phototransduction (Yamashita et al. 2008; Peirson et al. 2009; Shichida and Matsuyama 2009).

The phototransductive role of cerebellar encephalopsin would comply with the current opinion of cerebellar actions. Based on the immunohistochemical analyses, those cells with the highest fluorescence intensity between the granular and molecular layers of the cerebellar cortex sagittal section resemble Purkinje cell bodies. If it is true that GABA-ergic Purkinje cells are activated by illumination, the cascade would eventually lead up-state of the cerebral cortex, for example at the sensomotoric cortex, given that Purkinje cells have an inhibiting role between deep cerebellar nuclei and the cerebral cortex (Rowland et al. 2010). Furthermore, electrophysiological findings concerning light-triggered GABA-ergic inhibition of action potentials might also confirm this idea (Wade et al. 1988). Also of note is that cerebellum is known to cover a wide variety of responsibilities, ranging from motor functions to attention, executive control, language (human), visuo-spatial function and working memory (Stoodley and Schmahmann 2010), functions which are all emphasized during the waking state of the circadian rhythm.

Mouse PVA, containing hypothalamic nuclei, is the main part of the central area for neurosecretory regulation, thus being the main part of the homeostatic control mechanisms in vertebrates. Like actions in the human hypothalamus, mouse PVA neurons synthetizing trophic hormones release them via the capillaries of the portal system in the median eminence into the anterior pituitary (adenohypophysis), which secretes six physiologically significant hormones. The posterior pituitary (neurohypophysis) is the storage and release site for two neurohormones, oxytocin and vasopressin (known also antidiuretic hormone, ADH). The secretory neurons of the hypothalamus are innervated by monoaminergic neurons using serotonin (5-HT), dopamine (DA), and noradrenaline (NA) as neurotransmitters. Based on our results on the abundance of OPN3 in mouse PVA, there arises an interesting question of whether phototransduction via OPN3 directly modulates the neurosecretion of trophic hormones, and consequently, supports the adjustment of hormonal circadian rhythms.

The cerebral cortex in mice, as in all mammals, has a key role in cognitive functions, like memory, attention, perceptual awareness, thought, and consciousness. The putative role of OPN3-mediated phototransduction in these areas is impossible to cover here in detail, due to the enormous complexity and diversity of higher brain functions. Some preliminary ideas of enhanced cortical functions are still allowed, since G-protein mediated responses might include both spikes, or even more preferably, enhanced membrane potentials of targeted neurons, which might potentiate neural activity to maintain circadian rhythms.

Even though the exact role of the endogenous opsin-mediated phototransduction cascade in the mouse brain still remains elusive, literature describes direct extraretinal photosensitive responses to light in the rodent brain. Firstly, as a response to direct cortical illumination by ambient visible light, a release of inhibitory neurotransmitter gamma-aminobutyric acid (GABA), as well as electrophysiological changes in GABA-ergic currents, has been shown (Wade et al. 1988; Leszkiewicz and Aizenman 2003). In addition, Leszkiewicz et al. (2000) found in their patch-clamp study an enhanced *N*-methyl-d-aspartate (NMDA) receptor-mediated response in cortical neurons. Moreover, a wide variety of evidence exists for at least monochromatic light’s capability to trigger, for example, serotonin, dopamine, noradrenaline, and cytokines release (Shen-Zeng et al. 1982; Cassone et al. 1993). Finally, an excretion of corticosterone, as well as its clock gene activation, has been shown to be a response to monochromatic light (Rahman et al. 2011). Consequently, light seems to modulate neurotransmission through additional means other than opsin and G-protein mediated pathways alone. For example, the studies of Leszkiewicz and co-workers suggest that light regulates neuronal activity by direct allosteric modulation of GABA- and NMDA-receptor protein (Leszkiewicz et al. 2000; Leszkiewicz and Aizenman 2003).

In conclusion, the present study shows for the first time that OPN3 is expressed at the protein level in different brain areas of mice. The finding of extraocular OPN3 photoreceptor protein in the mammalian brain may support the idea of these proteins' putative roles in phototransductive functions. Given that opsins are known to mediate phototransduction (cf. Terakita 2005), and that OPN3 is suggested to play a role in non-visual photic processes (cf. Kasper et al. 2002; White et al. 2008), further investigations are needed to show the potential physiological regulation of OPN3 protein expression due to the changes in the ambient light. Also, to explore the exact cellular location of OPN3, an immuno-EM study is appropriate for further investigation.
